# Editorial: Reproducible analysis in neuroscience

**DOI:** 10.3389/fninf.2024.1520012

**Published:** 2024-11-26

**Authors:** Stavros I. Dimitriadis, Vignayanandam Ravindernath Muddapu, Roberto Guidotti

**Affiliations:** ^1^Department of Clinical Psychology and Psychobiology, Faculty of Psychology, University of Barcelona, Barcelona, Spain; ^2^Institut de Neurociències, University of Barcelona, Campus Mundet, Barcelona, Spain; ^3^Integrative Neuroimaging Lab, Thessaloniki, Greece; ^4^Cardiff University Brain Research Imaging Centre (CUBRIC), School of Psychology, College of Biomedical and Life Sciences, Cardiff University, Cardiff, United Kingdom; ^5^Blue Brain Project, École Polytechnique Fédérale de Lausanne (EPFL), Campus Biotech, Geneva, Switzerland; ^6^Department of Neuroscience, Imaging and Clinical Sciences, University “G. D'Annunzio” Chieti-Pescara, Chieti, Italy; ^7^Institute for Advanced Biomedical Technologies (ITAB), University “G. D'Annunzio” Chieti-Pescara, Chieti, Italy

**Keywords:** reproducible analysis, repeatability and reproducibility, R&R studies, neuroscience, neuroimaging (anatomic and functional), analytic pipelines, reproducibility of results

One of the key ingredients of scientific progress is the ability to repeat, replicate, and reproduce independently important scientific findings. Recently, independent groups failed to replicate the results of several experiments in various research areas, opening the so-called “reproducibility crisis.” The reasons behind these failures may be motivated by the excessive trust given to the results obtained by digital computers. Indeed, little attention was given to the implementation of a principal algorithm, and method or to the variation introduced by the use of different software, and hardware systems or to how difficult a finding can be recover after weeks or years or to the precision level one had performed a computational experiment (Donoho et al., 2009; Peng, 2011).

To extricate this tight tangled set of terms, it is important to precisely define the meaning of reproducing, replicating, and repeating with the terminology long established in experimental sciences (Plesser, 2018). The *Association for Computing Machinery* (ACM) has adopted the following definitions for the three highly used terms on research (Association for Computing Machinery, 2016).

Repeatability (Same team, same experimental setup, and same data): the measurements (findings) can be obtained with precision by the same team, using the same measurement procedure (experimental protocol), the same measuring system, under the same operating conditions (e.g., neuroimaging system like MRI 3T with the same set-up, time of the day, etc.), in the same location (Lab) following a multiple trial acquisition protocol. For solely computational experiments, it practically means that a researcher can reliably repeat his/her own computations.

Replicability (different team, same experimental setup, and different data): the measurements (findings) can be obtained with precision by a different team, using the same measurement procedure (experimental protocol), the same measuring system, under the same operating conditions (e.g., neuroimaging system like MRI 3T with the same set-up, time of the day, etc.), in the same location (Lab) following a multiple trial acquisition protocol. For solely computational experiments, it practically means that an independent group can obtain the same result by employing the author's own experimental artifacts.

Reproducibility (different team, different experimental setup, and different data): the measurements (findings) can be obtained with precision by a different team, using a different measurement procedure (experimental protocol), a different measuring system, under similar operating conditions (e.g., neuroimaging system like MRI 3T with the same set-up, time of the day, etc.) in a different location (Lab) following a multiple trial acquisition protocol. For solely computational experiments, it practically means that an independent group can obtain the same result using experimental artifacts produced completely independently from the author's artifacts.

For more information, an interested researcher can read informative studies discussing this terminology (Crook et al., 2013; Goodman et al., 2016; Nichols et al., 2017).

In computational neuroscience, there are two types of studies: simulation experiments and advanced analyses of experimental data. In both types of studies, methods reproducibility refers to obtaining the same results when running the same code again. However, this type of reproducibility demands access to experimental data, code, and simulation specifications (Botvinik-Nezer and Wager, 2023). Results reproducibility demands access to the experimental data, but the analysis can be realized by using different pipelines (combination of methods, code) e.g., analysis packages or neural simulators.

In the methodological part of a study, data analysis demands a lot of decisions. More recently, 70 independent analysis teams tested nine prespecified hypotheses using the same task–functional magnetic resonance imaging (fMRI) dataset (Botvinik-Nezer et al., 2020). The 70 teams selected 70 different analytical pipelines, and this variation affected the results, including the statistical maps and conclusions drawn regarding the preselected hypotheses tested. In a recent study, Luppi et al. (2024) systematically evaluated 768 data-processing pipelines for network construction from resting-state functional MRI, evaluating the effect of brain parcellation, global signal regression, and connectivity definition.

**Figure 1:**
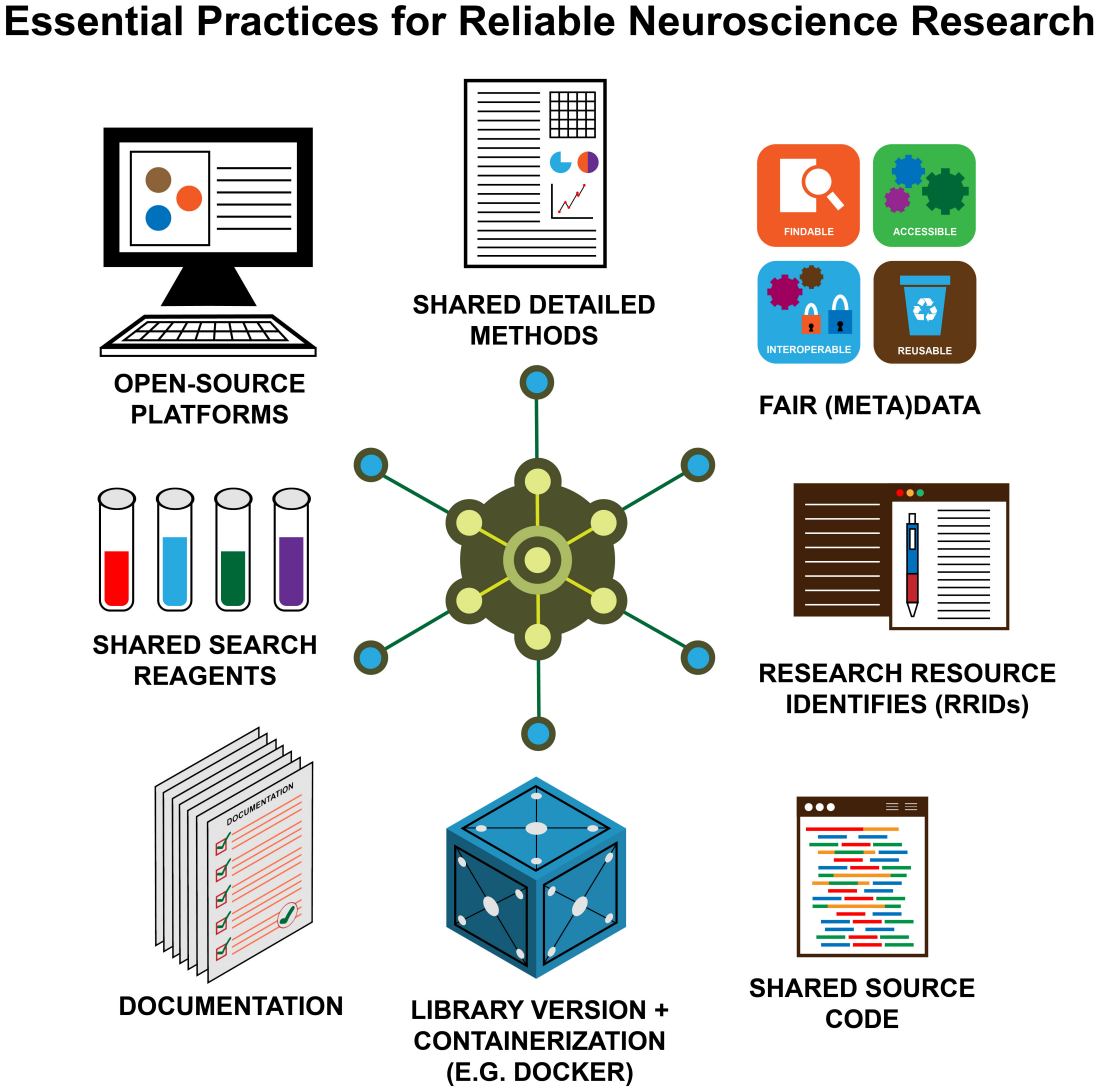
The key elements of reproducible (neuro)science. Open—source platforms; FAIR (meta)data; shared, detailed methods; shared source code; shared search reagents; documentation; research resource identifies (RRIDs); library version + containerization (e.g., Docker).

Several organizations worldwide have tried to increase awareness about the importance of reproducibility and replicability in different disciplines in recent years (e.g., www.repro4everyone.org, Global Reproducibility Networks), including open-source repositories for research resources (e.g., Zenodo), protocols, source code (e.g., GitHub), datasets, etc. Figure 1 illustrates schematically the key ingredients to make a research study reproducible and replicable (Auer et al., 2021).

In recent years, myriad tools have been developed to support rigor, reproducible, and replicable research findings. Another key term that the community adopted to support these practices is open science. Open science is made up of three big pillars: data, code, and articles (Gorgolewski and Poldrack, 2016).

In the data direction, several efforts have been made to build platforms to openly upload experimental data and organize it into a common and shareable format (e.g., BIDS). Code sharing is also crucial since open data should be processed with tools and scripts that are available and versioned. In this regard, the adoption of collaborative and versioning platforms such as GitHub has increased the accessibility of software specific to publication. Moreover, tools that automate and containerize software have reduced the problems of software versions and the burden of time to control it. Finally, articles should describe methods and data well and be published, if possible, in open-access journals or preprints to increase transparency.

In this Research Topic, we collected articles that push toward the direction of reproducibility and adopting open science practices in neuroscience.

Ioanas et al. focused on a new concept: reexecution. This concept, which is innovative in reproducibility studies, concerns the possibility to run the very same pipeline using the tools and data shared with the publication to reproduce the findings of a study. They presented an automated workflow for full, end-to-end article reexecution generating the full research communication output from the raw data, and automatically executable code. This study underlines the feasibility of article regeneration as a process that takes advantage of data and tools sharing in conjunction with containerization.

As we mentioned above, sharing data is crucial for reproducibility, and McPhee et al. discussed in a transparent way the different challenges of collecting, harmonizing, and analyzing data collected in a collaborative research network. They faced problems in the harmonization of data across different disorders, partners, and formats. Their proposed strategy overcame the aforementioned restrictions faced by other research groups and will further help the researchers understand the health research outcomes in children with neurodevelopmental disorders (NDDs).

Naseri et al. discussed the disparities between MRI and PET research in terms of scientific standards, analytic plan pre-registration, data and code sharing, containerized workflows, and standardized processing pipelines. They discussed the importance of the research community in PET to follow general practices of MRI research as a way to release the full potential of brain PET research.

Another important ingredient for open science and reproducibility is code. In addition to sharing the analysis pipelines, it is also important to build software that allows reproducibility. In this Research Topic, different articles propose their tools to ease transparency and reproducibility.

Winchester et al. demonstrated the Eventer website as a common framework to upload, analyze, and share the findings, including meta-data and supervised learning-assisted models as a way for enhancing reproducibility when analyzing datasets of spontaneous synaptic activity.

Meyers introduced a package that makes it easy to perform decoding neural analyses in the R programming language, implementing a range of different analytic pipelines. This new R package will help researchers create reproducible and shared decoding analyses.

Ister et al. introduced a MATLAB package called SuMRak. It integrates brain segmentation, volumetry, image registration, and parameter map generation into a unified interface, thereby reducing the number of separate tools that researchers may require for straightforward data handling. This package offers an efficient MRI data processing of preclinical brain images, enabling researchers to extract consistent and precise measurements, significantly reducing the operating time, and allowing the analysis of large datasets.

Routier et al. presented Clinica, an open-source software platform designed to make clinical neuroscience studies easier and reproducible. Clinica provides processing pipelines for MRI and PET images that involve the combination of different software packages, the transformation of input data to the Brain Imaging Data Structure (BIDS), and the store of the output data using the ClinicA Processed Structure (CAPS). This platform supports the reproducible analysis in neuroimaging research.

Ji et al. demonstrated the QuNex software tool, which is an integrative platform for reproducible multimodal neuroimaging analytics. It provides an end-to-end execution capability of the entire study neuroimaging workflow, from data onboarding to analyses, to be customized and executed via a single command. QuNex platform is optimized for high-performance computing (HPC) or cloud-based environments, enabling high-throughput parallel processing of large-scale open neuroimaging datasets. This platform supports the reproducible neuroimaging analysis.

In conclusion, the eight articles published in this Research Topic emphasize the strength and importance of replicating research studies to confirm and advance our knowledge in neuroscience. We would like to draw attention to reviewers when editing and reviewing replication research studies, as we have revealed that not every researcher understands the importance of publishing replication studies. An independent reviewer should always focus on the existence of the source code, and the availability of research data in open accessible repositories, and the study design as a minimal confirmation of the robustness and generalizability of novel findings. We hope this Research Topic and editorial will stimulate the research community to conduct more replication studies, following the important rules of reproducible neuroscience and understanding the importance and value of reproducibility and replicability in (neuro)science.
